# Dental implant treatment for two adjacent missing teeth in the esthetic region: A systematic review and 10‐year results of a prospective comparative pilot study

**DOI:** 10.1002/cre2.773

**Published:** 2023-08-17

**Authors:** Henny J. A. Meijer, Kees Stellingsma, Christiaan W. P. Pol, Arjan Vissink, Barzi Gareb, Gerry M. Raghoebar

**Affiliations:** ^1^ Department of Oral and Maxillofacial Surgery, University of Groningen University Medical Center Groningen Groningen The Netherlands; ^2^ Department of Restorative Dentistry, Dental School, University of Groningen University Medical Center Groningen Groningen The Netherlands

**Keywords:** adjacent implants, cantilever, esthetic zone, systematic review

## Abstract

**Objectives:**

The aim of the systematic review was to compare studies on implant‐supported two‐unit cantilever crowns with two adjacent implant‐supported crowns in the anterior region. The second aim was to assess in a 10‐year prospective comparative pilot study, hard and soft peri‐implant tissue changes in patients with a missing central and adjacent lateral upper incisor, treated with either an implant‐supported two‐unit cantilever crown or two single implant‐supported crowns.

**Materials and Methods:**

Medline, Embase, and the Cochrane Central Register of Controlled Trials were searched (last search March 1, 2023). Inclusion criteria were studies reporting outcomes of two missing adjacent teeth in the esthetic region and treated with a single implant‐supported two‐unit cantilever fixed dental prosthesis, or with two solitary implant‐supported crowns. Outcome measures assessed included implant survival (primary), changes in marginal bone and gingiva level, restoration survival, subjective and objective esthetic scores, papilla volume, mid‐facial marginal mucosa level, probing depth, bleeding on probing, and biological and technical complications with ≥1‐year follow‐up. In addition, in a 10‐year pilot study, the same outcome measures were assessed of five patients with a single implant‐supported two‐unit cantilever crown and compared with five patients with two adjacent single implant‐supported crowns in the esthetic zone.

**Results:**

Nine articles with 11 study groups were found eligible for data extraction. Meta‐analyses of implant survival rates were 96.9% (mean follow‐up 3.4 ± 1.4 years) for the implant‐cantilever treatment and 97.6% (mean follow‐up 3.0 ± 1.8 years) for the adjacent implants treatment (*p* = .79). In the 10‐year comparative pilot study, no clinically relevant changes in hard and soft peri‐implant tissue levels occurred in both groups. Patient satisfaction was also high in both groups.

**Conclusion:**

Single implant‐supported two‐unit crowns can be a viable alternative to the placement of two adjacent single implant crowns in the esthetic zone.

## 1 INTRODUCTION

Replacement of two missing adjacent teeth is considered a difficult treatment in implant dentistry (Van Nimwegen et al., 2019; Ramanauskaite & Sader, 2022). If these missing teeth are located in the esthetic zone, treatment becomes even more challenging due to the esthetic demands regarding the presence and stability of papillae adjacent to the implant restorations (Ramanauskaite & Sader, 2022). The presence of the papillae is determined predominantly by the attachment levels of the neighboring teeth (Ramanauskaite & Sader, 2022; Roccuzzo et al., 2018). When two adjacent teeth are missing in the esthetic zone, there is a tendency that the presence of the papilla between two implant crowns is determined by the inter‐implant bone crest distance (Ramanauskaite et al., 2018; Rivara et al., 2020). As a consequence, the reduced inter‐implant papilla height can lead to the development of a black triangle between two adjacent implant crowns, reducing the esthetic outcome.

In the case of a missing adjacent upper central incisor and a lateral incisor, there is often little horizontal space to place two adjacent implants, even when smaller diameter implants are used in the region of the lateral incisor. When placing two implants in this region, the implants are often placed too close to each other, and resorption of the inter‐implant bone crest leads to recession of the inter‐implant papilla (Ramanauskaite et al., 2018). An alternative is to place one implant in the region of the central incisor and an implant crown on this implant connected with a cantilever at the position of the lateral incisor. With this treatment, bone crest height is not affected by approximal resorption between adjacent implants. Van Nimwegen et al. (2017) conducted a systematic review of the clinical outcome of single implant‐supported two‐unit cantilever fixed denture prostheses. They concluded that two‐unit cantilever crowns could be a viable alternative to the placement of two adjacent single implant crowns in the esthetic zone. However, it was also mentioned that studies reporting on implant‐supported cantilever prostheses usually reported on three or four‐unit cantilever prostheses supported by two or more implants. Little was known about single implant‐supported two‐unit cantilever crowns in the esthetic zone and the literature reporting on this treatment consisted mostly of a short‐term evaluation. Therefore, the aim of the present study was to systematically review the literature on the outcome of single implant‐supported two‐unit cantilever crowns in the esthetic region. A second aim was to assess the 10‐year results of a prospective comparative pilot study of patients with a missing central and lateral upper incisor treated with either one implant and an implant crown with a cantilever or two implants with solitary implant crowns.

## 2 MATERIALS AND METHODS SYSTEMATIC REVIEW

### 2.1 Research question

The protocol for this review was registered at the National Institute for Health Research in the PROSPERO International Prospective Register of Systematic Reviews (ID408717). The following research question was formulated: “What are implant and restoration survival rates, changes in marginal bone and gingiva levels, subjective and objective esthetic scores, papilla index, probing depth, bleeding on probing scores, and biological and technical complications in patients with two missing adjacent teeth, being central incisors, lateral incisors, cuspids or first bicuspids and treated with a single implant‐supported two‐unit cantilever fixed dental prosthesis compared to two solitary implant‐supported crowns?.” Based on this research question, the following PICOS was deduced:

– **P**atients: patients with two missing adjacent teeth in the esthetic region, being central incisors, lateral incisors, cuspids, or first bicuspids;
– **I**ntervention: single implant‐supported two‐unit cantilever fixed dental prosthesis
– **C**ontrol: two solitary implant‐supported crowns;
– **O**utcomes: implant survival (primary), changes in marginal bone and gingiva level, restoration survival, subjective and objective esthetic scores, papilla volume, mid‐facial marginal mucosa level, probing depth, bleeding on probing and biological and technical complications with ≥ 1‐year follow‐up;
– **S**tudy designs: randomized controlled trials, prospective cohort and/or controlled studies, retrospective cohort and/or controlled studies, and case series (≥5 patients per group). Systematic reviews and animal studies were excluded.

There were no language restrictions. If studies were reported on the same study group, results with the longest follow‐up period were included.

An extensive search of the literature was conducted and was completed on March 1, 2023. The databases used were Medline (PubMed), Embase, and the Cochrane Central Register of Controlled Trials (CENTRAL). The search was completed by a hand search of the references found in eligible studies. The search strategy was developed in cooperation with a bioinformation specialist and adapted to the syntax rules of each database (Table 1).

**Table 1:** Search strategy in Medline (PubMed), Embase, and Cochrane Central Register of Controlled Trials (CENTRAL) and number of titles found.

PubMed
("Esthetics, Dental"[Mesh:NoExp] OR "Jaw"[Mesh] OR esthet*[tiab] OR aesthet*[tiab] OR cosmet*[tiab] OR maxill*[tiab] OR mandib*[tiab] OR ((front*[tiab] OR anterior*[tiab] OR incisor*[tiab] OR canine*[tiab]) AND (dent*[tiab] OR tooth[tiab] OR teeth[tiab] OR crown*[tiab])))
AND
("Dental Implantation"[Mesh] OR "Dental Implants"[Mesh] OR implant*[tiab])
AND
(cantilever*[tiab] OR “adjacent implant*”[tiab] OR “adjacent missing”[tiab] OR “missing adjacent”[tiab] OR “two‐unit*”[tiab] OR “2‐unit*”[tiab])
NOT
(("Animals"[Mesh] NOT "Humans"[Mesh]) OR "Letter" [pt])
*529 titles*
Embase (embase.com) ('maxilla'/exp OR 'mandible'/exp OR 'incisor'/exp OR 'canine tooth'/de OR 'maxillary canine'/exp OR (esthet* OR aesthet* OR cosmet* OR maxill* OR mandib* OR ((front* OR anterior* OR incisor* OR canine*) AND (dent* OR tooth OR teeth OR crown*))):ab,ti,kw)
AND
('tooth implant'/exp OR 'tooth implantation'/exp OR implant*:ab,ti,kw)
AND
('cantilever'/exp OR cantilever* OR ((adjacent NEAR/2 (implant* OR missing)) OR ‘two‐unit*’ OR ‘2‐unit*’):ab,ti,kw)
NOT
(('animal'/exp NOT 'human'/exp) OR 'letter'/exp OR 'conference abstract'/it)
*625 titles*
Cochrane CENTRAL (Trials)
([mh ^"Esthetics, Dental"] OR [mh Maxilla] OR [mh Mandible] OR (esthet* OR aesthet* OR cosmet* OR maxill* OR mandib* OR ((front* OR anterior* OR incisor* OR canine*) AND (dent* OR tooth OR teeth OR crown*))):ti,ab,kw)
AND
([mh "Dental Implantation"] OR [mh "Dental Implants"] OR implant*:ti,ab,kw)
AND
(cantilever* OR (adjacent NEAR/2 (implant* OR missing)) OR "two‐unit" OR "2‐unit"):ab,ti,kw
*63 titles (Trials)*

### 2.2 Study selection

Two reviewers (Henny J. A. Meijer and Gerry M. Raghoebar) independently screened the results from the electronic searches, according to the inclusion and exclusion criteria as defined in the PICOS elements, in two rounds. Articles were first screened by title and abstract. In case of a positive result or in case of doubt, the study was moved to the next round (full‐text assessment). Any disagreement regarding the inclusion was resolved by a consensus discussion. In case of persistent disagreement, an external independent reviewer (Kees Stellingsma) with experience in implant dentistry was consulted.

### 2.3 Quality assessment

Case series were assessed by the same two reviewers (Henny J. A. Meijer and Gerry M. Raghoebar) with the Newcastle–Ottawa scale (NOS) (Wells et al., 2000), and each article was rated from 0 to 9 stars for each parameter in the scale. Studies scoring ≥6 stars were considered to be high in methodological quality, while <6 stars indicated low quality. If being a randomized controlled trial, the Cochrane risk of bias tool RoB 2.0 (Sterne et al., 2019) was used and the ROBINS‐1 tool (Sterne et al., 2016) for prospective clinical non‐randomized trials. Any disagreement was resolved by consensus between the reviewers.

### 2.4 Statistical analysis

Inter‐observer agreement was calculated as Cohen's *κ* and percentage of agreement. A qualitative synthesis of the included studies was performed. A meta‐analysis was carried out on implant survival rate if the studies were sufficiently homogenous (*I* ^2^ < 50%). The subgroups consisted of patients with two‐adjacent implant‐supported restorations and patients with single implant‐supported two‐unit cantilever fixed dental prostheses. A random‐effects model with the DerSimonian–Laird estimator was used to calculate and compare the mean implant survival rate with 95% confidence intervals (95% CIs) between subgroups. Heterogeneity was expressed as *I* ^2^.

## 3 MATERIALS AND METHODS COMPARATIVE CLINICAL STUDY

### 3.1 Patient selection

A comparative pilot study with two parallel groups was designed and has been described in detail reporting 1‐year results (Tymstra et al., 2011) and 5‐year results (Van Nimwegen et al., 2017) and therefore briefly described. Patients were referred to University Medical Center Groningen (Groningen, the Netherlands) for implant treatment of two missing adjacent anterior maxillary teeth. Between January 2005 and February 2008 10 consecutive patients fulfilling the inclusion criteria were registered. This 10‐year evaluation was not considered to be clinical research with test subjects, as meant in the Medical Research Involving Human Subjects Act (WMO) (METc communication M22.293344) and registered in ClinicalTrials.gov (ID 202200150). Patients were informed about collecting data for the 10‐year evaluation and all patients gave informed consent. The study was conducted in accordance with the 2008 revised requirements of the Helsinki Declaration of 1975 and reported following the PRISMA and STROBE guidelines.

**Figure 1:**
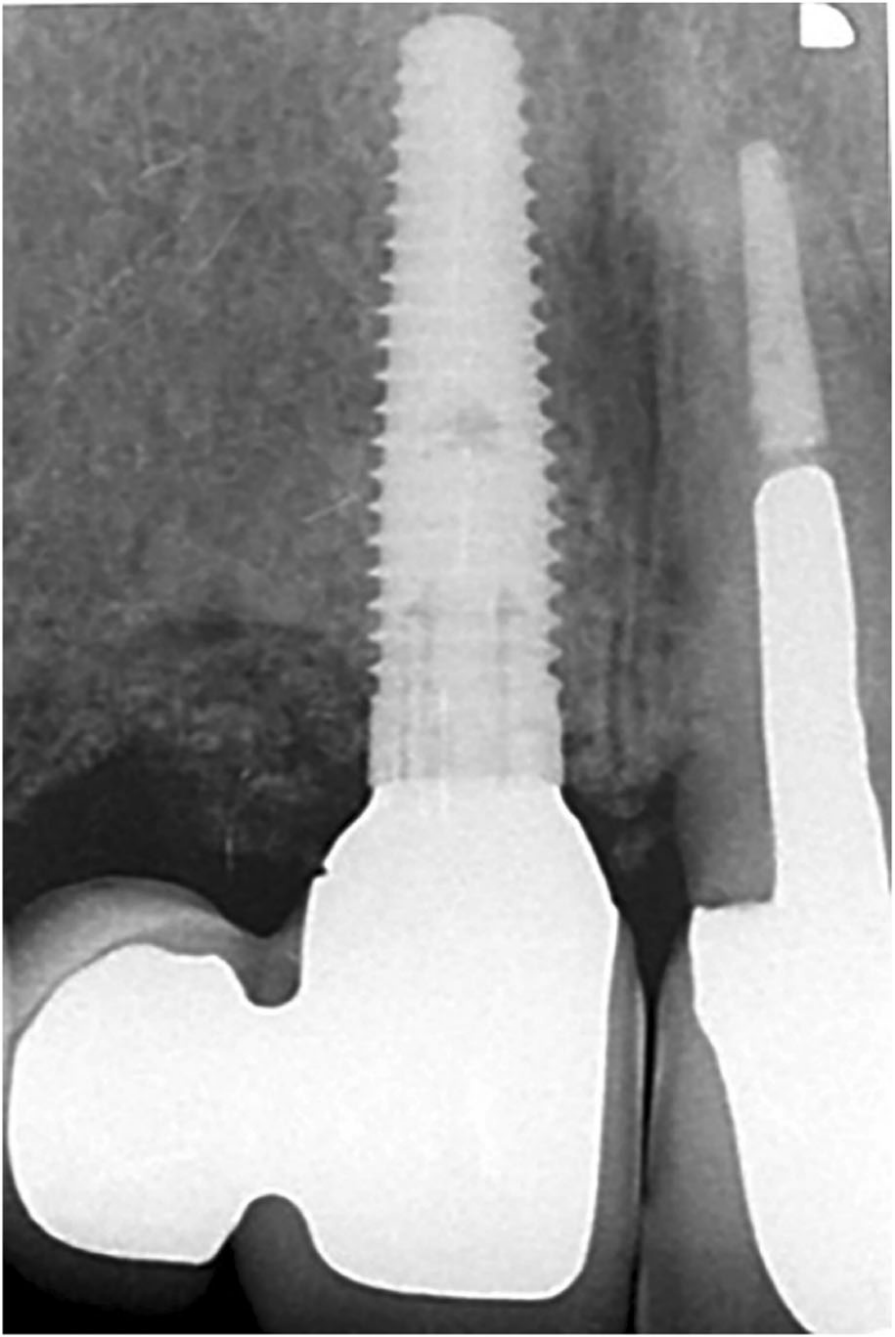
Ten‐years radiograph of implant‐supported two‐unit cantilever crown.

**Figure 2:**
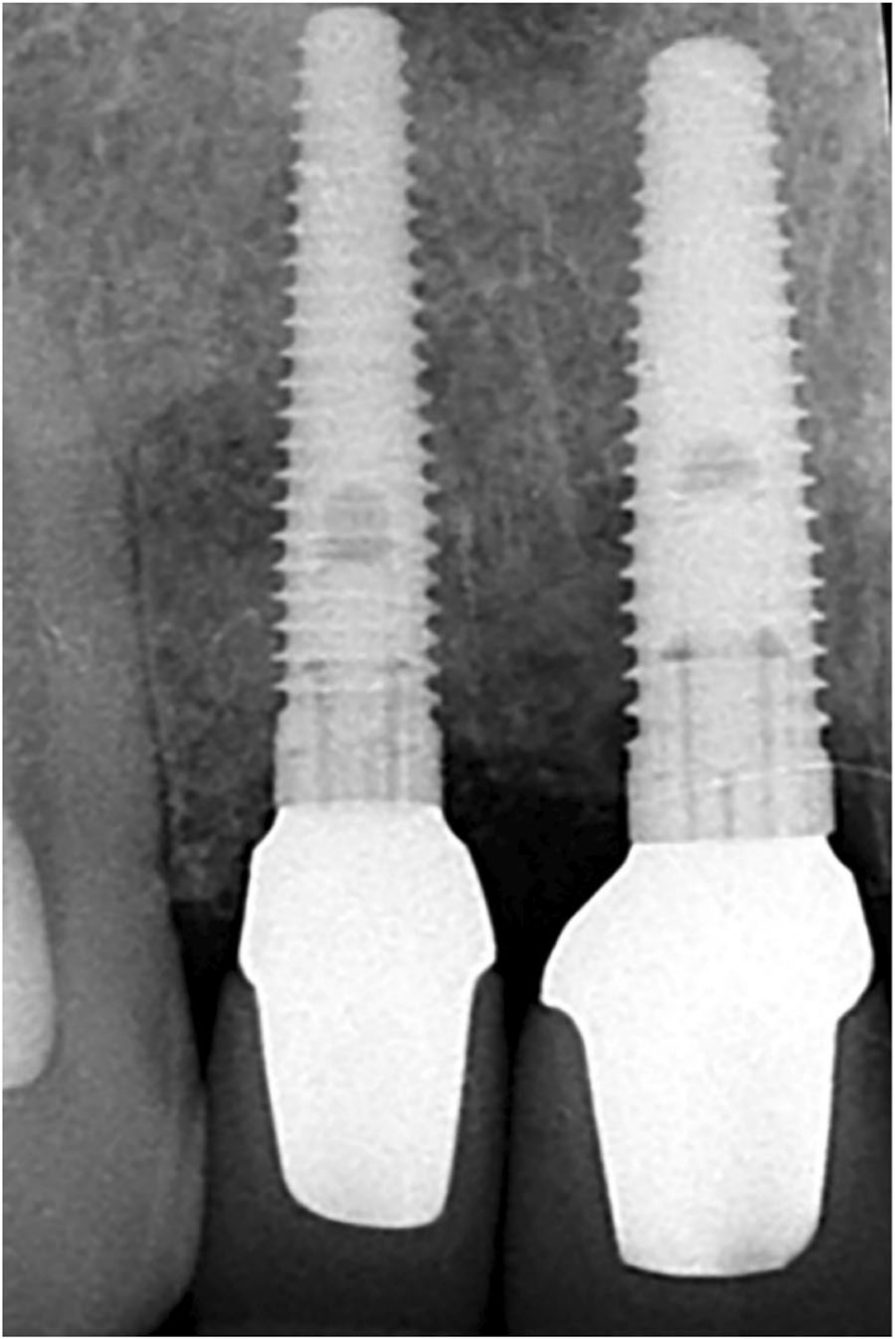
Ten‐years radiograph of two adjacent implant‐supported crowns.

The study population was divided into two groups:

(1) “Implant‐cantilever group”: Five patients to treat with one dental implant in the region of the central incisor (NobelReplace Groovy Regular Platform; Nobel Biocare AB, Göteborg, Sweden); prosthetic restoration will consist of an implant crown connected with a cantilever at the position of the lateral incisor (Figure 1).
(2) “Implant‐implant group”: Five patients to treat with two adjacent dental implants (NobelReplace Groovy Regular Platform at the position of the central incisor and NobelReplace Groovy Narrow Platform at the position of the lateral incisor); prosthetic restoration will consist of two single‐tooth implant crowns (Figure 2).

### 3.2 Data collection

Reporting of data was at baseline, directly after placement of the definitive implant crown (T0) and 10 years after placement of the definitive crown (T10). The following parameters were assessed:

– Implant and restoration loss during the entire evaluation period;
– Marginal bone level and bone crest level change;
– Mid‐facial marginal soft‐tissue level change;
– Papilla Index according to Jemt (1997);
– Implant probing depth;
– Modified Bleeding Index according to Mombelli et al. (1987);
– Gingiva Index according to Löe and Silness (1963);
– Esthetic outcome (Pink Esthetic score/White Esthetic Score) as described by Belser et al. (2009);
– A subjective appreciation of the final result of the treatment was assessed using a patient questionnaire modified from the one used by Meijndert et al. (2004);
– Technical complications.

### 3.3 Statistical analysis

The statistical analysis has been restricted to means and standard deviation due to the pilot study design with small groups.

## 4 RESULTS SYSTEMATIC REVIEW

**Figure 3:**
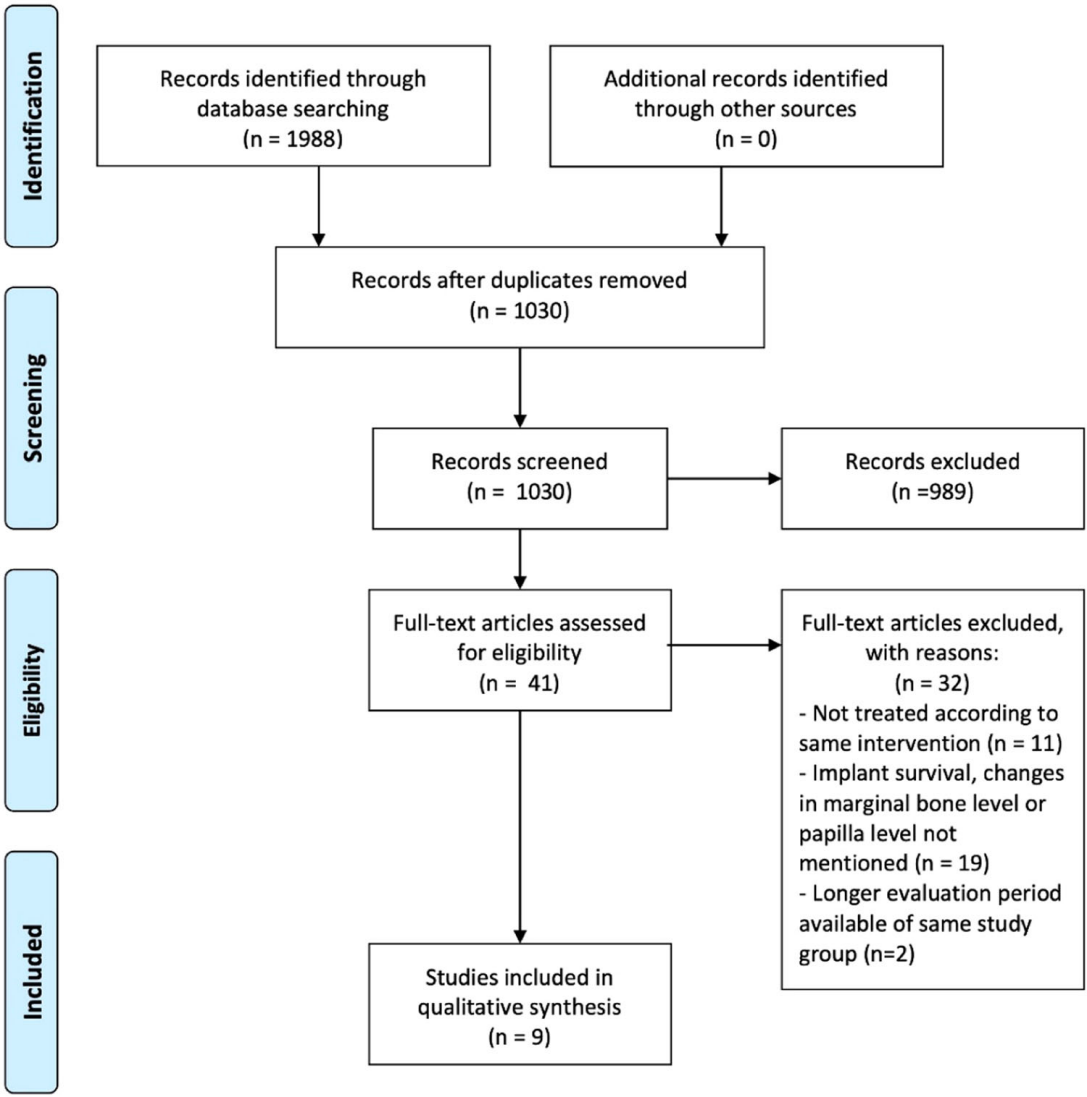
Algorithm of the study selection procedure.

The results of the primary search were 529 hits for the Medline search, 625 hits for the Embase search, and 63 for the Cochrane Library search (last search March 1, 2023). After the exclusion of double titles, a total of 1030 papers were identified for further screening. After scanning of titles and abstracts, 989 articles were excluded and 41 articles were selected for full‐text evaluation. After full‐text evaluation, another 32 articles were excluded. A total of nine articles (with 11 study groups) fulfilling the inclusion criteria were selected for qualitative assessment and data extraction. There was substantial agreement between the two reviewers' judgments (*κ* = 0.767, 98.2% agreement) at title/abstract selection. At full‐text selection, there was no disagreement between the reviewers (*κ* = 1.0, 100% agreement). There was no need to consult the third reviewer in any of the study selection phases. The algorithm of the study selection procedure is depicted in Figure 3. When an article reported data from two groups, the groups were viewed separately. Consequently, a total of three groups (mean follow‐up 3.4 ± 1.4 years; 57 implants) had an implant‐cantilever construction, and eight groups (mean follow‐up 3.0 ± 1.8 years; 298 implants) had two adjacent implants. Characteristics and outcomes of the included studies are described in Tables 2 and 3. Risk‐of‐bias with the Newcastle–Ottawa quality assessment scale revealed that 10 studies were judged as having high methodological quality (91%) and one study had a low methodological quality (Table 4). Because of the heterogeneity in study designs and data presented, the outcomes of the studies are mainly presented as a descriptive review. However, all studies reported on implant survival rate leading to the possibility of conducting a meta‐regression analysis on this outcome.

**Table 2:** Study characteristics and outcomes of included studies with single implant‐supported two‐unit cantilever fixed dental prostheses.

Study	Study design	Follow‐up (years)	Implant brand/type	No. of implants	Maxilla/mandibula	Implant survival (%)	Peri‐implant marginal bone level change (mean ± SD in mm)
Wu et al. (2013)	Retrospective case series	Mean 2.5 (1.3–3.7)	Straumann/not specified (NS) and NobelBiocare/NobelReplace	33	Mandible	100	Cantilever side −1.46 ± 0.53
							Noncantilever side −1.23 ± 0.43
Van Nimwegen et al. (2017)	Prospective case series	5	NobelBiocare/NobelReplace	5	Maxilla	100	Cantilever side −2.06 ± 1.51
							Noncantilever side −1.01 ± 0.74
Roccuzzo et al. (2020)	Retrospective case series	Mean 2.8 ± 1.6	Straumann/Straumann Bone Level and Dentsply Sirona/Astra Osseospeed	19	Maxilla and mandible	100	Cantilever side −0.1 ± 0.9
							Noncantilever side −0.1 ± 1.0

Study	Papilla^a^	Mid‐facial marginal soft‐tissue level change	Implant probing depth (mean ± SD in mm)	Bleeding Index	Gingiva Index	Esthetic outcome rated by professionals	Patient satisfaction	Restoration survival (%)	Technical complications
Wu et al. (2013)	Level change −0.22 ± 0.41 mm	Not reported (NR)	2.4 ± 1.6	No: 20.5%	NR	NR	mean 8.9 (range 7−10) (scale 0−10)	100	Screw loosening *n* = 1
				Mild: 40.1%					
				Moderate: 32.6%					
				Severe: 6.8%					
Van Nimwegen et al. (2017)	Index score^b^	NR	Cantilever side 3.8 ± 0.7	NR	NR	NR	9.2 ± 0.4 (scale 0−10)	NR	NR
	0: 80%		Non‐cantilever side 3.2 ± 0.5						
	1: 20%								
	2: 0%								
	3: 0%								
Roccuzzo et al. (2020)	Index score^b^	NR	No probing depths ≥5 mm	NR	NR	Anatomic form score: 63% excellent	NR	100	No complications
	0: 0%								
	1: 5%					Crown color match score: 58% excellent			
	2: 63%								
	3: 32%					Marginal adaption score: 69% no misfit			

^a^ Papilla between the implant crown and the cantilever crown;

^b^ Index according to Jemt or modified into Index according to Jemt (1997).

**Table 3:** Study characteristics and outcomes of included studies with two adjacent implant‐supported restorations.

Study	Study design	Follow‐up (years)	Implant brand/type	No. of implants	Maxilla/mandibula	Inter‐implant distance (mean ± SD in mm)	Implant survival (%)	Peri‐implant marginal bone level change (mean ± SD in mm)
Grossberg (2001)	Prospective case series	1	NobelBiocare/Brånemark	24	Maxilla	NR	100	NR
Tymstra et al. (2010)	Retrospective case series	Mean 4.3 (range 1.6–8.5)	NobelBiocare/Brånemark	20	Maxilla	3.0 ± 1.3	100	NR
Siqueira et al. (2013)	Retrospective case series	Range 0.5–5	NS	58	Maxilla	NR	100	NR
Van Nimwegen et al. (2015)	Prospective case series	5	NobelBiocare/NobelPerfect and NobelReplace	80	Maxilla	3.6 ± 1.1	97.5	Implant side −1.4 ± 0.9
								Tooth side −1.5 ± 0.7
Van Nimwegen et al. (2017)	Prospective case series	5	NobelBiocare/NobelReplace	10	Maxilla	NR	100	Implant side −1.4 ± 0.8
								Tooth side −1.4 ± 0.8
Van Nimwegen et al. (2019)	Prospective case series	1	NobelBiocare/NobelActive	32	Maxilla	NR	100	Implant side −0.1 ± 0.5
								Tooth side +0.1 ± 0.3
Rivara et al. (2020)	Prospective case series	1	NobelBiocare/NobelReplace	60	Maxilla and mandible	2.5	100	NS
Roccuzzo et al. (2020)	Retrospective case series	Mean 4.0 ± 3.0	Straumann/Straumann Bone Level and Dentsply Sirona/Astra Osseospeed	14	Maxilla and mandible	2.6 ± 0.7	93	Implant side −0.1 ± 0.9
								Tooth side −0.1 ± 1.0

Study	Papilla^a^	Mid‐facial marginal soft‐tissue level change	Implant probing depth (mean ± SD in mm)	Bleeding Index	Gingiva Index	Esthetic outcome rated by professionals	Patient satisfaction	Restoration survival rate (%)	Technical complications
Grossberg (2001)	Mean level change −0.4 mm	Not reported (NR)	NR	NR	NR	NR	NR	NR	NR
Tymstra et al. (2010)	Index score^b^ 0: 40% 1: 50% 2: 0% 3: 10%	NR	Implant side 3.4 ± 0.9 Tooth side 3.7 ± 1.0	Index score^c^ 0: 20% 1: 35% 2: 45% 3: 0%	Index score^d^ 0: 50% 1: 55% 2: 0% 3: 0%	Implant Crown esthetic Index 70% acceptable result	8.3 ± 0.8 (scale 0–10)	NR	NR
Siqueira et al. (2013)	Index score^b^ 0 and 1: 66% 2 and 3: 34%	NR	NR	NR	NR	NR	NR	NR	NR
Van Nimwegen et al. (2015)	Index score^b^ 0: 26% 1: 54% 2: 17% 3: 3%	NR	Implant side 4.4 ± 1.6 Tooth side 4.0 ± 1.8	Index score^c^ 0: 37% 1: 34% 2: 26% 3: 3%	Index score^d^ 0: 80% 1: 17% 2: 0% 3: 0%	Implant Crown esthetic Index majority poor result	8.8 ± 1.3 (scale 0–10)	NR	NR
Van Nimwegen et al. (2017)	Index score^b^ 0: 20% 1: 60% 2: 20% 3: 0%	NR	Implant side 3.3 ± 1.3 Tooth side 2.8 ± 1.0	NR	NR	NR	9.0 ± 1.0 (scale 0–10)	NR	NR
Van Nimwegen et al. (2019)	Index score^b^ mean 0.48 ± 0.53	NR	Implant side 2.9 ± 0.7 Tooth side 2.6 ± 0.6	Not specified (NS)	NS	Modified Pink Esthetic Score/White Esthetic Score (PES/WES) 13.0 ± 2.1	9.3 ± 0.7 (scale 0–10)	100	No complications
Rivara et al. (2020)	Index score^b^ 0: 3% 1: 40% 2: 57% 3: 0%	–0.5 ± 0.9	NR	NR	NR	NR	NR	NR	NR
Roccuzzo et al. (2020)	Index score^b^ 0: 0% 1: 14% 2: 57% 3: 26%	NR	No probing depths ≥5 mm	NR	NR	Anatomic form score: 60% excellent Crown color match score: 38% excellent Marginal adaption score: 53% no misfit	NR	93	No complications

^a^ Papilla between the implant crown and the cantilever crown.

^b^ Index according to Jemt or modified into Index according to Jemt (1997).

^c^ Modified Bleeding Index according to Mombelli et al. (1987).

^d^ Gingiva Index according to Löe and Silness (1963).

**Table 4:** Risk‐of‐bias of included case series (Newcastle‐Ottawa quality assessment).

Study	Group	Selection				Comparability		Outcome			Total score
		Exposed cohort	Nonexposed cohort	Ascertainment of exposure	Outcome of interest	Most important factor	Additional factor	Assessment of outcome	Length of follow‐up	Adequacy of follow‐up	
Wu et al. (2013)	Cantilever	*		*	*		*	*	*		6
Van Nimwegen et al. (2017)	Cantilever	*	*	*	*		*	*	*	*	8
Roccuzzo et al. (2020)	Cantilever	*	*	*	*		*	*	*		7
Grossberg (2001)	Two restorations	*		*	*		*	*		*	6
Tymstra et al. (2010)	Two restorations	*		*	*		*	*	*	*	7
Siqueira et al. (2013)	Two restorations	*		*	*		*	*			5
Van Nimwegen et al. (2015)	Two restorations	*		*	*	*	*	*	*	*	8
Van Nimwegen et al. (2017)	Two restorations	*	*	*	*		*	*	*	*	8
Van Nimwegen et al. (2019)	Two restorations	*		*	*		*	*		*	6
Rivara et al. (2020)	Two restorations	*		*	*		*	*		*	6
Roccuzzo et al. (2020)	Two restorations	*	*	*	*		*	*	*		7

*Note*: Studies with ≥6 stars are considered to be high in methodological quality, and ˂6 stars indicate low quality.

### 4.1 Implant survival

**Figure 4:**
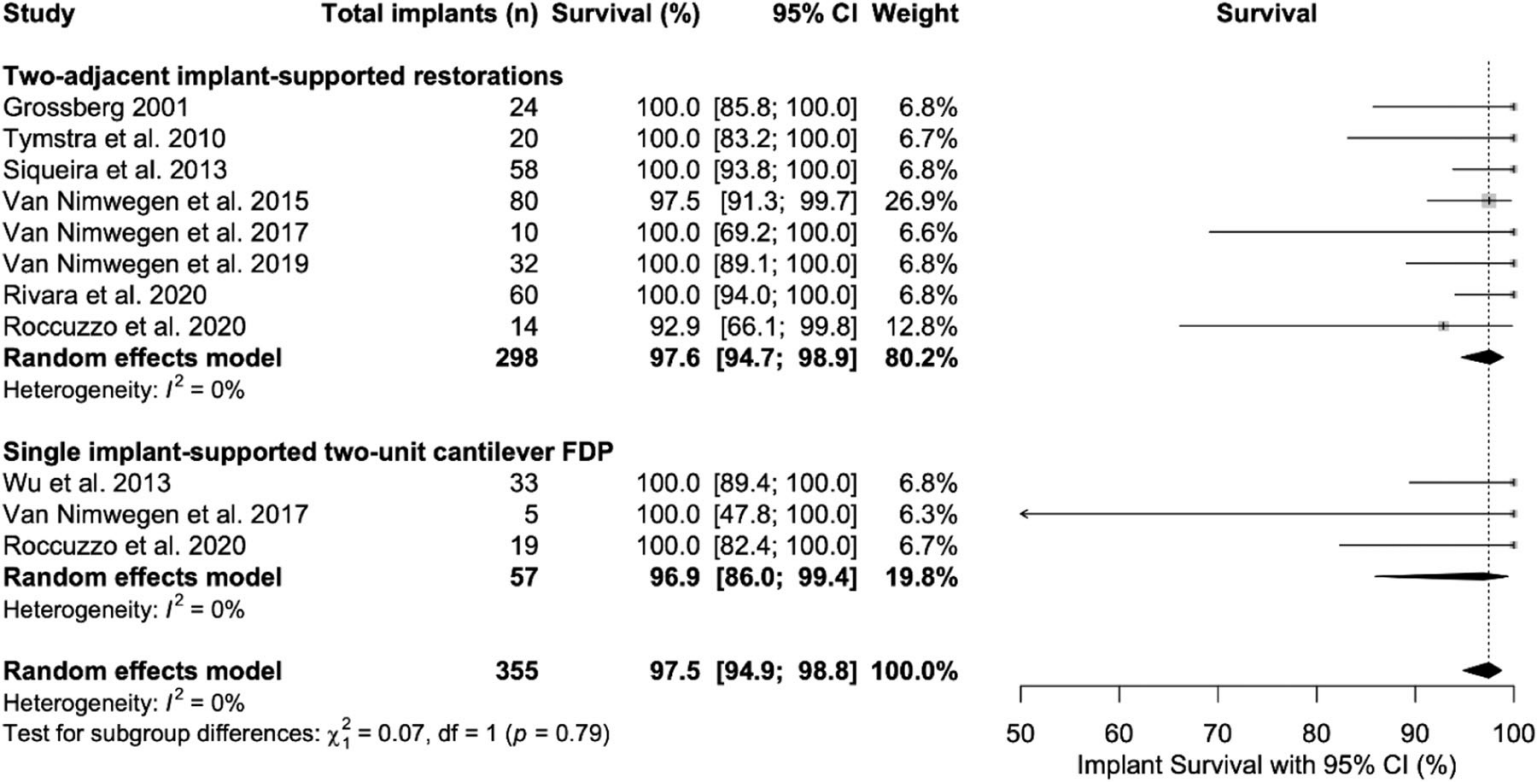
Forest plot for random effects meta‐analysis of studies evaluating implant survival rate.

All 11 study groups reported on implant survival. The implant survival rate in the Implant‐cantilever and implant‐implant groups were 96.9 (95% CI: 86.0–99.4) after a mean follow‐up of 3.4 ± 1.4 years and 97.6 (95% CI: 94.7–98.9) after a mean follow‐up of 3.0 ± 1.8 years, respectively (Figure 4). The subgroup analysis revealed no significant statistical difference in implant survival between the subgroups (*p* = .79).

### 4.2 Marginal bone level changes

Mean marginal bone changes of the Implant‐Cantilever groups are described in Table 2. Mean marginal bone loss at the cantilever side varied from 0.1 ± 0.5 mm after 1 year (Roccuzzo et al., 2020) to 2.06 ± 1.51 mm after 5 years (Van Nimwegen et al., 2017). Mean marginal bone changes of the Implant‐Implant groups are described in Table 3. Mean marginal bone loss at the inter‐implant side varied from 0.1 ± 0.5 mm after 2.8 years (Van Nimwegen et al., 2019) to 1.4 ± 0.8 mm after 5 years (Van Nimwegen et al., 2017).

### 4.3 Papilla volume

Papilla volume was recorded in all studies, mostly using the Papilla Index according to Jemt (1997).

### 4.4 Mid‐facial marginal soft‐tissue level change

The mid‐facial marginal soft‐tissue level change was only mentioned in the study of Rivara et al. (2020) on two adjacent implants, revealing a recession of 0.5 ± 0.9 mm after 1 year.

### 4.5 Implant probing depth

Implant probing depths were measured in 8 out of the 11 studies, being mostly of limited depth.

### 4.6 Bleeding‐Index and Gingiva‐Index

The Bleeding Index and Gingiva Index as an expression of peri‐implant soft tissue health were hardly mentioned in the studies.

### 4.7 Esthetic outcome rated by professionals

Esthetic outcome rated by professionals was only reported by Roccuzzo et al. (2020), Van Nimwegen et al. (2015), and Van Nimwegen et al. (2019), but in all three studies a different index was used.

### 4.8 Patient satisfaction

Patient satisfaction was rated in 6 of the 11 studies. They all used a scale of 0–10 and mean patient satisfaction was high in all studies.

### 4.9 Restoration survival rate and technical complications

Restoration survival rate and technical complications were hardly rated in the included studies.

## 5 RESULTS COMPARATIVE PILOT STUDY

All 10 patients could be evaluated during the 10‐year evaluation period. No implants and no restorations were lost, resulting in a 100% survival rate. Mean (SD) of marginal bone level change (mm), mean (SD) of bone crest level change (mm), and mid‐facial marginal soft‐tissue level change (SD) during the 10‐year evaluation period are listed in Table 5. Bone loss and soft tissue recession are minor in both groups. The frequency distribution of Papilla Index scores at 10 years is listed in Table 6. Papilla presence is compromised at the implant‐cantilever site and the inter‐implant site. The mean pocket probing depth, mean Bleeding Index, and mean Gingival Index are listed in Table 7. Probing depths are limited in both groups and peri‐implant soft tissues are healthy. Esthetic outcome (Pink Esthetic score/White Esthetic Score) as described by Belser et al. (2009) has been described in Table 8. Pink Esthetic scores are relatively low in both groups. Overall patients' satisfaction is high in the Implant‐cantilever group (9.4 ± 0.9) as well as in the Implant‐implant group (9.0 ± 1.0) (Table 9). Technical complications during the 10‐year follow‐up were limited to twice chipping (polishing, no repair needed) and once loosening of the restoration (re‐cemented) in the Implant‐cantilever group. Technical complications in the Implant‐implant group were limited to chipping in one patient (polishing, no repair needed) and a mobile restoration in another patient (screw retightened).

**Table 5:** Mean (SD) of marginal bone level change (mm), mean (SD) of bone crest level change (mm) and mid‐facial marginal soft‐tissue level change (SD) in mm 10 years after placement of the restoration.

		Implant‐cantilever	Implant‐implant
*Bone level change*		T10	T10
Central implant	Marginal bone level facing the adjacent central incisor	−0.11 (0.24)	−0.13 (0.28)
	Marginal bone level facing no implant/lateral implant	−0.37 (0.42)	−0.12 (0.27)
Bone crest	Bone crest level between the central implant and no implant/lateral implant	−0.15 (0.33)	−0.16 (0.12)
No implant/lateral implant	Marginal bone level facing the adjacent central implant	Not applicable (NA)	−0.02 (0.05)
	Marginal bone level facing the adjacent cuspid	NA	0.00 (0.00)
*Soft tissue level change*		T10	T10
Central implant	Mid‐facial marginal soft‐tissue level change	0.97 (1.17)	0.37 (0.85)
No implant/lateral implant	Mid‐facial marginal soft‐tissue level change	NA	0.31 (0.76)

**Table 6:** Frequency distribution of scores of Papilla Index up to 10 years after placement of the definitive restoration.

	Implant‐cantilever						Implant‐implant					
	Central tooth‐implant		Implant‐cantilever		Cantilever‐cuspid		Central tooth‐implant		Implant‐implant		Implant‐cuspid	
Score	T0	T10	T0	T10	T0	T10	T0	T10	T0	T10	T0	T10
0	0	0	2	0	0	0	0	0	2	0	0	0
1	0	1	3	5	1	1	4	1	3	3	0	1
2	3	2	0	0	2	2	1	4	0	2	4	1
3	2	2	0	0	2	2	0	0	0	0	1	3
4	0	0	0	0	0	0	0	0	0	0	0	0

*Note*: Score 0, no papilla formation; score 1, less than half of the papilla present; score 2, at least half of the papilla is present; score 3, papilla fills whole approximate space; score 4, abundance of papilla. T0, evaluation visit directly after placement of definitive restoration; T10, evaluation visit 10 years after placement of definitive restoration.

**Table 7:** Mean and standard deviation (SD) of pocket probing depth (mm) (measured around implants at the proximal sides facing the adjacent implant, mid buccally and the proximal sides facing the adjacent tooth), mean Bleeding Index (SD), and Gingival Index (SD) of peri‐implant mucosa at baseline (T0) and 10 years after placement.

		Implant‐cantilever		Implant‐implant	
		T0	T10	T0	T10
Central implant	Probing depth proximal side facing adjacent tooth	3.4 (0.89)	3.0 (0.0)	2.2 (0.84)	2.4 (0.5)
	Probing depth midbuccally	3.0 (0.71)	3.6 (0.9)	2.2 (0.84)	2.8 (0.4)
	Probing depth proximal side facing no implant/lateral implant	4.4 (0.89)	3.4 (1.1)	2.4 (0.55)	3.6 (0.5)
	Bleeding Index (possible score 0–3)	1.2 (0.4)	0.6 (0.9)	1.4 (0.5)	1.2 (0.4)
	Gingival Index (possible score 0–3)	0.4 (0.9)	0.4 (0.9)	0.6 (0.5)	0.4 (0.5)
No implant/lateral implant	Probing depth proximal side facing adjacent tooth	Not applicable (NA)	NA	3.2 (1.6)	2.6 (0.5)
	Probing depth midbuccally	NA	NA	3.0 (1.0)	3.2 (0.8)
	Probing depth proximal side facing no implant/lateral implant	NA	NA	2.8 (0.5)	3.2 (0.8)
	Bleeding Index (possible score 0–3)	NA	NA	1.0 (0.0)	0.6 (0.5)
	Gingival Index (possible score 0–3)	NA	NA	0.4 (0.5)	0.2 (0.4)

*Note*: T0, evaluation visit directly after placement of definitive restoration; T10, evaluation visit 10 years after placement of definitive crown.

**Table 8:** Mean values (SD) of esthetic evaluation (Pink Esthetic score/White Esthetic Score [PES/WES]) at the 10‐year follow‐up.

	Central implant		Lateral cantilever	
	T0	T10	T0	T10
**Implant‐cantilever group**				
PES	5.0 (0.7)	4.8 (0.8)	5.4 (2.1)	5.6 (2.2)
WES	9.4 (0.5)	8.2 (1.3)	8.0 (1.2)	8.4 (1.5)
PES/WES	14.4 (1.1)	13.0 (1.9)	13.4 (3.1)	14.0 (3.4)
**Implant‐implant group**				
PES	4.8 (0.8)	6.6 (0.9)	4.4 (1.3)	6.6 (1.7)
WES	7.4 (0.9)	7.8 (1.3)	7.4 (1.5)	7.0 (2.0)
PES/WES	12.2 (0.4)	14.4 (2.1)	11.8 (1.9)	13.0 (3.8)

**Table 9:** Mean (SD) scores on patient satisfaction questionnaires at the 10‐year follow‐up.

	Implant‐cantilever	Implant‐implant
	T10	T10
Shape of the restoration	3.6 (0.5)	3.6 (0.5)
Color of the crown	3.4 (0.9)	4.0 (0.0)
Shape of the mucosa	2.6 (1.1)	3.6 (0.5)
Color of the mucosa	2.8 (1.6)	4.0 (0.0)
Overall score (range 0–10)	9.4 (0.9)	9.0 (1.0)

*Note*: Implant restoration and mucosa score: scale 0, completely dissatisfied; 1, dissatisfied; 2, neutral; 3, satisfied; 4, completely satisfied. Overall score: scale 0, completely dissatisfied to score 10, completely satisfied.

## 6 DISCUSSION SYSTEMATIC REVIEW

This systematic review attempted to assess the outcome of single implant‐supported two‐unit cantilever crowns and two adjacent implant‐supported restorations in the esthetic region of the maxilla and mandible. Only three studies were reported on cantilever crowns and eight studies on adjacent crowns. This can be considered very limited given the difficulty of treatment in a challenging esthetic region (Ramanauskaite & Sader, 2022). One would expect more publications to share outcomes and possible guidelines for a better treatment prognosis. Next to this, the follow‐up period did not exceed 5 years. No long‐term studies could be included. In addition, most studies did not report a full‐scale outcome focussing on clinical outcomes, radiographical outcomes, and patient satisfaction. The reason was, for example, that some studies were focussing on soft tissue response after treatment and other studies solely on the influence of inter‐implant distance. Thus, a meta‐analysis was only meaningful for implant survival rate as this was the only similar outcome reported in all studies. The implant survival rate was high and without a significant difference between the Implant‐cantilever and Implant‐implant groups. This high implant survival rate is comparable with single‐implant survival rates in the esthetic region (Bassir et al., 2019). Apparently, dental implant treatment in the esthetic region in case of two adjacent missing teeth does not lead to a lower implant survival rate compared to single tooth treatment, irrespective of the implant‐based treatment procedure.

Marginal bone level changes varied widely and even exceeded 1 mm in some studies, especially at the cantilever side and at the sides facing another implant. Because of the small number of implants placed in the anterior maxilla and mandibula, no conclusions can be drawn from these studies, but the absence of a natural tooth with the attachment of connective tissue fibers seems to have a negative impact on peri‐implant bone levels. In line with this finding, papilla scores seem to be compromised between the crown and cantilever and between two adjacent restorations. Also on papilla volume, the absence of a natural tooth with attachment of connective tissue fibers to seems have a negative impact. The mid‐facial marginal soft‐tissue level change was only reported in one study (Rivara et al., 2020), which is remarkable for studies in the esthetic region. Implant probing depths were measured in 8 out of the 11 studies, being mostly of limited depth. It suggests that the presence of a two‐unit cantilever crown might not affect peri‐implant probing depths. Bleeding on probing and gingival infection were hardly reported (Van Nimwegen et al., 2015; Tymstra et al., 2010; Wu et al., 2013) which is remarkable because they are important prognostic outcome measures for peri‐implant health. Esthetic outcomes rated by professionals were only reported by Roccuzzo et al. (2020), Van Nimwegen et al. (2015, 2019), but in all three studies a different index was used. Consensus conferences are needed to define a uniform set of outcome measures to evaluate dental implant treatment. Most studies used a scale of 0–10 to score patient satisfaction and mean satisfaction was high in both treatment groups. Restoration survival rate and technical complications were hardly rated in the included studies. It could be that there were no complications, but reporting is important in light of patient satisfaction and economic implications.

## 7 DISCUSSION COMPARATIVE PILOT STUDY

Two adjacent implant‐supported restorations and one implant with restoration with the cantilever in the maxillary esthetic region showed a high implant and restoration survival rate, stable peri‐implant bone and mid‐facial mucosa levels, healthy peri‐implant soft tissues and a high patients' satisfaction during 10 years of follow‐up. Papillae between two single implant‐supported restorations or between an implant‐supported restoration and cantilever were scored as insufficient, as well as the pink esthetics scored by professionals.

Results of the present 10‐year follow‐up study could best be compared with other comparative long‐term studies on the same topic, but these long‐term studies are missing. Two medium‐term studies could be found, Van Nimwegen et al. (2017) and Roccuzzo et al. (2020), but it must be noted that the study group of Van Nimwegen et al. (2017) is the same as in the present study. Therefore, the comparison has been made with the retrospective comparative case series of Roccuzzo et al. (2020) with 19 patients with an implant‐cantilever restoration (mean follow‐up 2.8 ± 1.6 years) and with 14 patients having two adjacent implant‐supported restorations (mean follow‐up 4.0 ± 3.0 years) in the esthetic region of the upper or lower jaw.

Implant and restoration survival rates were 100% in the Implant‐cantilever group in the study of Roccuzzo et al. (2020). The implant and restoration survival rate was 93% in the Implant‐implant group (one implant and one restoration were lost) in the study of Roccuzzo et al. (2020). In the present study, no implants and restorations were lost during 10 years of follow‐up in both groups, resulting in a survival rate of 100%, which is more or less similar. The mean marginal bone level change at both sides (either facing the tooth side or the cantilever side/implant side) of the implants was −0.1 mm. Also in the present study, the peri‐implant bone level was very stable with bone loss in the same order of magnitude. The mean recession of mid‐facial marginal mucosa was not exceeding 1 mm in both groups in the present study; this recession is minor during the 10‐year evaluation period. Mid‐facial marginal soft tissue level was not reported in the study of Roccuzzo et al. (2020). Comparing papilla presence in both groups, in the study of Roccuzzo et al. (2020) as well as in the present study, it seems that the papilla is more compromised between cantilever and implant restoration and between two implant restorations than at the side that faces the natural tooth. However, it must be noted that in the present study, these papillae seem more compromised than in the study of Roccuzzo et al. (2020). In the present study, it was shown that this was not only the case at the final follow‐up evaluation but also already at baseline. The attachment of connective tissue fibers at the surface of a natural tooth root is an important prerequisite for maintaining papilla volume. Between implants or at the implant side facing the cantilever there are no connective tissue fibers helping to maintain or develop a full papilla. Probing depths are limited in both groups and peri‐implant soft tissues are healthy. This finding corresponds with the findings of Roccuzzo et al. (2020). It must be mentioned that both studies were university‐based, meaning a strict inclusion protocol and strict follow‐up protocol with respect to oral hygiene procedures and reinstruction. It must be noted that probing depth measurements at implant sites have a lower degree of reliability compared to natural teeth. Measurements at implant sites are influenced by implant type, force applied during probing, depth of probing, pain during probing, and design of the restoration (Ramanauskaite et al., 2023). Both studies used a different esthetic outcome rated by professionals, making comparison difficult. In the present study, the Pink Esthetic Score/White Esthetic Score (PES/WES) was used. The PES/WES was rated for the majority of patients of both groups as having moderate esthetics, especially of the soft tissues. One can conclude that professionals rated the result far from excellent and more or less moderate, especially for the soft tissues. Apparently, the history of trauma with extended bone loss and difficulties to maintain papilla volume does have an impact on the final appearance. Patient satisfaction was not mentioned in the study of Roccuzzo et al. (2020). Patient satisfaction was very high in the present study, with no differences between both groups. Although Papilla‐Index scores pointed to compromised papillae in both groups, this did not seem to have a negative effect on patient satisfaction. This is comparable to the findings of other studies (Meijndert et al., 2007; Van Nimwegen et al., 2015). Roccuzzo et al. (2020) mentioned that no technical complications occurred during the evaluation period. Technical complications during the 10‐year evaluation were limited and reparable in the present study. Apparently, the materials used and the connection between components are strong enough to survive a considerable period of function.

## 8 CONCLUSIONS

The data shown from the included studies in the systematic review indicate that single implant‐supported two‐unit cantilever crowns as well as two adjacent implant‐supported crowns show good implant survival rates on short‐term to midterm and excellent patient satisfaction. Data on soft‐tissue levels are lacking or insufficient. Thus, no conclusions can be made regarding these outcomes. In the 10‐year prospective comparative pilot study, no large differences in hard‐ and soft‐tissue levels and patient satisfaction could be shown between patients with a missing central and lateral upper incisor treated with either one implant and an implant crown with a cantilever or two implants with solitary implant crowns. However, a limitation is the limited number of patients in the comparative study. But, within this limitation of the study, a single implant‐supported cantilever crown in the esthetic zone can be a viable alternative to the placement of two adjacent single implant crowns.

## AUTHOR CONTRIBUTIONS

**Henny J. A. Meijer**: Concept/design; data collection; data analysis/interpretation; drafting article and approval of article. **Kees Stellingsma**: Data collection; critical revision of article and approval of article. **Christiaan W. P. Pol**: Critical revision of article and approval of article. **Arjan Vissink**: Data analysis/interpretation; critical revision of article and approval of article. **Barzi Gareb**: Data collection; critical revision of article and approval of article. **Gerry M. Raghoebar**: Concept/design; critical revision of article and approval of article.

## CONFLICT OF INTEREST STATEMENT

The authors declare no conflict of interest.

## Data Availability

Data that support the findings of this study are available upon reasonable request from the corresponding author. The data are not publicly available due to privacy or ethical restrictions. Data are available on request due to privacy/ethical restrictions.
